# In Vitro Human Gastrointestinal Digestibility and Colonic Fermentation of Wheat Sourdough and Yeast Breads

**DOI:** 10.3390/foods13183014

**Published:** 2024-09-23

**Authors:** Ccori Martinez Tuppia, Mohammad N. Rezaei, François Machuron, Cindy Duysburgh, Jonas Ghyselinck, Massimo Marzorati, Jonna E. B. Koper, Céline Monnet, Nabil Bosco

**Affiliations:** 1 Lesaffre Institute of Science and Technology, 59700 Marcq-en-Barœul, France; ccorimartinez@gmail.com (C.M.T.); m.rezaei@lesaffre.com (M.N.R.); f.machuron@lesaffre.com (F.M.); j.koper@lesaffre.com (J.E.B.K.); c.monnet@lesaffre.com (C.M.); 2 Prodigest, Technologiepark 82, 9052 Zwijnaarde, Belgium; cindy.duysburgh@prodigest.eu (C.D.); jonas.ghyselinck@prodigest.eu (J.G.); massimo.marzorati@prodigest.eu (M.M.)

**Keywords:** colonic fermentation, gut microbiome, in vitro digestibility, nutrient bio-accessibility, wheat bread

## Abstract

Bread can vary in textural and nutritional attributes based on differences in the bread making process (e.g., flour type, fermentation agent, fermentation time). Four bread recipes (BRs) made with sourdough preferments (BR1, white flour; BR2, whole grain flour) or regular yeast breads (BR3, white flour; BR4, whole grain flour) were evaluated for texture, digestibility, and their effect on the metabolic activity and composition of the gut microbiota using texture profile analysis (TPA) coupled with in vitro upper gastrointestinal (GIT) digestion and colonic fermentation (Colon-on-a-plate™ model), using fecal samples from eight healthy human donors. TPA revealed significantly higher values for hardness, fracturability, gumminess, and chewiness, and significantly lower values for springiness, cohesiveness, and resilience with whole grain versus white breads (all *p* < 0.001); values for springiness, cohesiveness, and resilience were significantly higher for sourdough versus yeast bread (*p* < 0.001). Nutrient composition and bioaccessibility were generally comparable between sourdough and yeast bread with similar flours. Following simulation of upper GIT digestion, all BRs demonstrated good digestibility of minerals, carbohydrates, and proteins. Colonic fermentation revealed changes in gut microbiota composition, significant increases in short-chain fatty acids, and a significant decrease in branched short-chain fatty acids with all BRs versus a blank. Overall, new insights into wheat bread digestibility and colonic fermentation were provided, which are important aspects to fully characterize bread nutritional profile and potential.

## 1. Introduction

Wheat bread, a staple in the human diet, provides multiple nutrients including carbohydrates, dietary fiber, minerals (e.g., iron, calcium, potassium), vitamins (e.g., thiamine, riboflavin, niacin, folate), and proteins [1]. Choices made during the breadmaking process, such as type of wheat, fermentation agent, and fermentation time, can impact both bread quality and nutritional composition [2]. On average, 100 g of commercial whole-wheat bread contains 12.3 g protein, 39.2 g carbohydrate (4.41 g sugar, 6 g fiber, 28.7 g starch), 163 mg Ca, 2.56 mg Fe, 76.6 mg Mg, 0.391 mg thiamine, and 4.43 mg niacin [3]. The optimal enrichment of nutrients in bread is important for maintaining the health of the human population [2]. Furthermore, the digestibility of nutrients is an important consideration when developing food products, as this indicates nutrient bioavailability, which may not always correlate with the nutrient content of a given food product.

In vitro models that simulate gastrointestinal digestion by mimicking both the upper gastrointestinal tract (GIT) and colonic conditions are widely used to study food digestibility. Such models can both complement in vivo studies and overcome some of their limitations (e.g., the inability to exclude interfering host effects, ethical concerns, and costs) [4,5]. Given their ability to provide relevant information on the rate and extent of nutrient digestion, a growing interest in using in vitro approaches to describe the digestibility of starch and proteins in foods has emerged [6,7,8]. Such information can be used as an indicator to manipulate the food matrix or processing conditions to improve digestibility [1,9]. For example, Khrisanapant et al. found that increased hydrothermal processing time improved the digestibility of starch and proteins in three types of legumes [9]. The influence of added ingredients can also be explored using this approach. Indeed, Johnston et al. used an in vitro digestion model to evaluate the kinetics of protein and starch digestibility of breads in which wheat flour was substituted with various amounts of whole-bean flour [1]. In that study, they found that breads with whole bean flour had reduced starch digestion kinetics and increased the rate of protein digestion.

In addition to bread digestibility and nutrient release during upper GIT digestion, the undigested fraction of bread can influence the human gut microbiome [10], which plays a pivotal role in both health and disease [5,11]. The composition of the gut microbiome and its derived metabolites (e.g., short-chain fatty acids [SCFA], amino acids, amino acid derivatives) impacts gut barrier function and host immune function [12,13]. Fermentation is one of the key modes of microbial metabolism in the gut. The fermentation process produces energy by metabolizing macromolecules (e.g., polysaccharides, amino acids) into chemicals that participate in microbe–microbe and microbe–host interactions [14]. Consequently, there is an increase in nutritional research strategies to modulate the gut microbiota to improve human health [5]. Gut microbiota-derived metabolites are primarily produced by colonic microorganisms during the fermentation of dietary fibers, proteins, and peptides that have resisted upper GIT digestion [15]. SCFAs are the most common of these microbial metabolites and include acetate, propionate, and butyrate in a 3:1:1 molar ratio [13]. In addition to SCFAs, which are beneficial to the human host, protein fermentation produces metabolites that can be toxic (e.g., phenol, p-cresol) [16].

The effects of bread intake on the gut microbiome have been investigated using in vitro fecal batch fermentation models [17,18,19]. For example, in vitro digestion and fecal fermentation of sourdough bread made with Tritordeum flour resulted in increased relative abundances of several bacterial genera including *Bifidobacterium* and increased production of acetate and butyrate [19]. A study investigating the effects of different bread processing methods on the gut microbiota reported that high carbohydrate utilization in sourdough bread fermentation was associated with increased butyrate production [20]. In another study, the fermentation of sourdough and yeast breads was compared using the Twin Mucosal-Simulator of the Human Intestinal Microbial Ecosystem (Twin M-SHIME^®^) model [18]. After two weeks of repeated administration, no significant changes in the composition of the luminal and mucosal microbiota were observed during the fermentation of the two bread types. However, at the metabolic level, the production of SCFAs (acetate, propionate, butyrate), isovaleric acid, 2-methylbutyric acid, and several amino acids (aspartate, threonine, glutamate, and γ-aminobutyric acid) significantly increased following the fermentation of sourdough bread compared to yeast bread. Evaluation of the effects of different breads on the production of microbial metabolites in the colon is important given that they could modulate host immune responses [21].

To our knowledge, there is a lack of data describing in vitro bread digestibility cautiously combining upper GIT digestion together with colonic fermentation. The aim of this study was to assess the in vitro digestibility of four bread recipes (BRs) made with white flour sourdough preferments (BR1); whole grain flour sourdough preferments (BR2); regular white flour yeast bread (BR3) and whole grain flour yeast bread (BR4). In addition, the impact of BR1–4 bread digestates on the composition and metabolic activity of the colonic microbiota was characterized.

## 2. Materials and Methods

**Figure 1:**
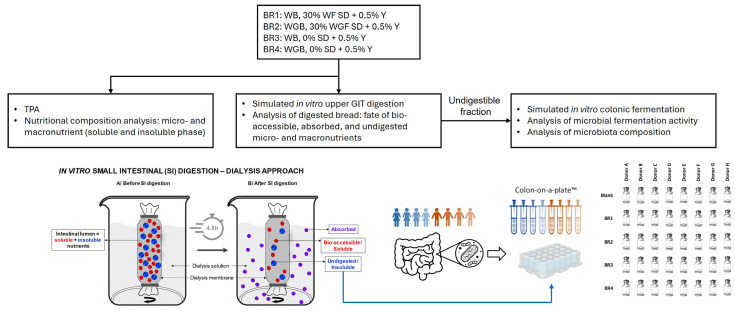
Experimental design. BR = bread recipe; Y = yeast; GIT = gastrointestinal tract; SD = sourdough; TPA = texture profile analysis; WB = white bread; WGB = whole grain bread; WF = wheat flour; WGF = whole grain flour.

This bread digestibility study included texture profile analysis (TPA), in vitro upper GIT digestion, analysis of the nutritional composition (pre- and post-digestion), and in vitro colonic fermentation using the Colon on-a-plate model^TM^, including an analysis of microbial fermentation activity and microbiota composition. The study design is summarized in Figure 1.

### 2.1. Flour Characterization

White and whole grain flours, milled from the same wheat kernels and without additives, were purchased from Moulins Waast (Mons-en-Pévèle, France). The following parameters were analyzed using American Association of Cereal Chemists (AACC)-approved methods: moisture content (AACC 44-15.02), ash content (AACC 08-01.01), falling number (AACC 56-81.03), and water absorption (%) (AACC 54-60.01) [22]. Protein content was determined using an automated Dumas protein analysis system (EAS, VarioMax N/CN, Elt, Gouda, The Netherlands), according to method 990.03 from the Association of Official Agricultural Chemists International (AOAC) [23].

### 2.2. Sourdough and Bread Preparation

Sourdough preferments were prepared on a 100 g flour basis by mixing flour (BR 1, white flour; BR2, whole grain flour) with water (BR1, 54%; BR2, 58%), salt (1.5%), and Livendo^®^ LV4 (*Levilactobacillus brevis*, *Saccharomyces chevalieri*) (Lesaffre International, Marcq-en-Barœul, France) (0.5%). The sourdough preferments were stored in a fermentation cabinet (30 °C, 90% relative humidity) and allowed to ferment for 24 h. In the final recipe (also on a 100 g flour basis), 30% of the corresponding sourdough was added to reach the final dough composition, which also included water (BR1, 65%; BR2, 72%), salt (2%), and compressed yeast (L’hirondelle bleue, Lesaffre International, Marcq-en-Baroeul, France) (0.5%). Sourdough preferments for BR1 and BR2 were prepared using the same flour type as in the final recipes.

BR3 and BR4 were prepared on a 100 g flour basis by mixing flour (BR3, white flour; BR4, whole grain flour) with water (BR3, 65%; BR4, 72%), salt (2%), and compressed yeast (L’hirondelle bleue, Lesaffre International) (0.5%).

The dough samples obtained from all recipes were divided into 320 g pieces, placed in baking pans, fermented in a proofing cabinet (35 °C, 90% relative humidity) for 180 min, and then baked for 22 min at 205 °C. The loaves of bread were then cooled for 2 h. For the digestibility study, bread samples were collected and stored at −20 °C. For TPA, bread samples were packaged in a plastic bag overnight and analyzed after 18 h.

All breadmaking experiments were performed in triplicate.

### 2.3. Bread TPA

TPA was performed using a texture analyzer (Stable Micro Systems Ltd., TA.XTplus, Godalming, UK) equipped with a P/25 probe. The hardness, springiness, cohesiveness, chewiness, gumminess, and resilience properties of the bread were analyzed as described by Kowalski et al. [24,25]. The test speed was 1 mm/s, and the compression rate was 60% of the sample height. The interval between the first and second compressions was 5 s, and the trigger force was 5 g.

### 2.4. In Vitro Upper GIT Digestion

The protocol used for the in vitro bread digestion was based on the consensus protocol developed within a large European network (COST Action InfoGest) [26]. This protocol included oral, gastric, and small intestinal incubation and was improved by utilizing more accurate pH profiles and including a simulation of small intestinal absorption using dialysis to remove molecules <3.5 kDa from the intestinal digests, as described by Van den Abbeele et al. [27]. For the oral incubation, 14 g of dry mass was mixed in a 1:2 (*w*/*v*) ratio with simulated salivary medium (as described by Mackie, et al. [26]) supplemented with α-amylase (750 U/mL, Sigma Aldrich, Merck KGaA, Darmstadt, Germany) and 105 µL 0.3 M CaCl_2_ solution, and it was then homogenized with a stomacher for 2 min. The samples were then incubated for 1 h (37 °C with shaking) in gastric medium (0.5 M hydrochloric acid was added to immediately reduce the pH from 5.5 to 2.0), using nutrient-depleted gastric fluid (7 mM KCl, 50 mM NaCl, 0.17 mM phosphatidylcholine, 4000 U/mL pepsin [Chem Lab, Zedelgem, Belgium]). The difference in water content between the different bread recipes was also corrected at this point. The pH was then increased from 2.0 to 5.5 using 1 M sodium bicarbonate, and the small intestinal incubation was initiated by adding 10 mM bile extract, 15.4 TAME U/mL trypsin (Carl Roth, Karlsruhe, Germany), 3.8 BTEE U/mL chymotrypsin (Carl Roth), 112.5 U/mL lipase (from porcine pancreas, Sigma Aldrich), 75 U/mL hog α-amylase (Sigma Aldrich), and 350 μL 0.3 M CaCl_2_ solution and incubating for 30 min (37 °C with mixing) to simulate the duodenal fraction. Small intestinal absorption was then simulated using a dialysis tube (ZelluTrans/Roth dialysis membrane, regenerated cellulose, molecular weight cut off 3.5 kDa) submerged in dialysis fluid (3.75 g/L NaHCO_3_; pH 7.0) for 4.5 h at pH 7). The dialysis fluid was collected (and pooled) and replaced every 45 min. The pooled dialysis fluid as well as the luminal content (sampled at the end of the small intestinal incubation period) were used to determine nutritional composition. The pooled dialysis fluid (unfiltered) was considered the absorbed fraction. The luminal content was further processed by centrifugation (5 min, 12,000× *g*). The soluble portion was filtered (0.2 µm filter) and considered the bio-accessible portion, while the insoluble portion was considered the undigested portion (Figure 1).

### 2.5. Nutritional Composition Pre- and Post-Digestion

Bread samples collected prior to upper GIT digestion were assessed for mineral, carbohydrate, and protein content. Samples collected after upper GIT digestion were assessed for mineral, carbohydrate, protein, amino acid, and antinutritional factor (phytate) content in the absorbed (dialysis fluid), bio-accessible (soluble intestinal content), and undigested (insoluble intestinal content) fractions.

#### 2.5.1. Mineral Quantification

Destruction of the undigested samples (i.e., bread samples prior to upper GIT digestion and the undigested fraction following upper GIT digestion) was performed using the closed microwave technique, in which 9 mL HNO_3_ was added to 1 g of sample followed by sample destruction at 180 °C for 2 h using a closed microwave. Samples were cooled, and ultra-pure water was added to 50 mL. For the soluble samples (i.e., absorbed and bio-accessible fractions collected after upper GIT digestion), 3.5 mL of HNO_3_ was added to 1 g of the sample and the mixture was incubated for 2 h. Ultra-pure water was then added to 20 mL. Mineral concentration (Ca, Mg, Fe, Zn, P) was then assessed by inductive coupled plasma-mass spectrometry (ICP-MS) in KED mode using a NexION 300 ICP-MS (PerkinElmer, Waltham, MA, USA).

#### 2.5.2. Carbohydrate Quantification

Carbohydrate concentrations were assessed using High-Performance Anion Exchange Chromatography with Pulsed Amperometric Detection (HPAEC-PAD). Bread samples prior to upper GIT digestion were assessed for free monomeric carbohydrates (glucose, maltose, fructose, galactose), as were the bio-accessible (intestinal lumen) and absorbed (pooled dialysis) fractions post-digestion. The concentration of non-resistant starch was determined using a commercial kit K-TSTA (Megazyme International Ireland, Bray, Ireland) through a hydrolysis step to quantify the glucose and maltose content of both pre-digested bread and the bio-accessible fraction after upper GIT digestion.

Briefly, HPAEC-PAD was performed using the Dionex ICS-3000 system (Thermo Fischer Scientific), using the CarboPac PA20 column (150 × 3 mm). The eluents for gradient analysis of the different monosaccharides included ultra-pure water (A), 100 M NaOH (B), and 1 M NaOAc in 100 mM NaOH (C). At the start, the isocratic phase was 10 min in 90% A and 10% B, followed by a linear gradient towards 100% B in 4 min. The second isocratic phase was 2 min in 100% B, followed by a linear gradient towards 80% B and 20% C in the first 6 min, towards 100% B in the next 2 min, and back to 90% A and 10% B in the final 2 min. The last isocratic phase was 6 min in 90% A and 10% B, in which the column was stabilized before the next injection. The total analysis time was thus 32 min with the flow rate set at 0.5 mL/min and a column temperature of 30 °C. Rhamnose (25 µM) was added to the samples before the HPAEC-PAD analyses as an internal standard to allow for accurate quantification. Monosaccharide quantification was further performed using the standards of the respective monosaccharides and recalculated based on peak areas.

#### 2.5.3. Protein Quantification

The degree of protein digestion was quantified via total nitrogen concentration according to the micro-Kjeldahl method [28]. The total nitrogen content was assessed for pre-digested bread and the luminal digesta (bio-accessible and undigested fractions). The absorbed fraction was determined by subtracting the digested protein from the total protein content.

Free amino acid composition in the absorbed fraction was determined by diluting the sample in 20% trichloroacetic acid (1:1 ratio) and incubating it for 2 h at 4 °C to allow for protein precipitation. Next, the samples were centrifuged (15 min; 15,000× *g*) and the supernatants were collected and dried with N_2_ gas. The resulting pellet was reconstituted in 900 µL ultra-pure water and 100 µL internal standard (25 mM alpha-aminobutyric acid), and the free amino acids were derivatized using 6-aminoquinolyl-N-hydroxysuccinimidyl carbamate to transform the primary and secondary amines into highly stable fluorescent derivatives. An Acquity^®^Arc Premier™ with a 2475 FLR detector (Waters, Milford, MA, USA) was used as a reverse-phase ultra-high performance liquid chromatography system to analyze the samples using an AccQ-tag-based method. An AccQ-tag Ultra C18 column (pore size 130 Å, particle size 2.5 µm, 4.6 mm i.d. × 100 mm, Waters) in combination with eluent A and B from the AccQ Tag Ultra-derivatization kit was used for separation. A gradient with a mobile phase (eluents A, B, and C [ultra-pure water]) was used. The non-linear separation gradient was 0–0.36 min (A, 2.0%; C, 98.0%), 19.67 min (A, 9.0%; B, 8.0%; C, 83.0%), 25.07 min (A, 8.0%; B, 17.6%; C, 74.4%), 28.20–30.08 min (A, 4.0%; B, 59.7%; C, 36.3%), 30.38–35.48 min (A, 2.0%; C, 98.0%). For each sample, 2 µL was injected for analysis. Excitation and emission wavelengths of 266 nm and 473 nm, respectively, were set on the fluorescence detector.

#### 2.5.4. Phytate Quantification

The concentration of phytate, an antinutritional factor, was determined in post-digestion samples using a commercial phytic acid/total phosphorus kit K-PHYT (Megazyme International Ireland, Bray, Ireland) according to manufacturer’s instructions, with some modifications. Briefly, dried digesta (bio-accessible fraction, 1 mL; absorbed fraction, 2.5 mL) was extracted in 1 mL HCl (0.66 M) with overnight incubation (room temperature, 100 rpm shaking). This was followed by enzymatic dephosphorylation and colorimetric determination of the phosphorus concentration. Measured concentrations of phosphorus were then used to determine the phytate concentration according to the manufacturer’s instructions (the measured phosphorus is exclusively released from phytic acid and comprises 28.2% of the phytic acid concentration).

### 2.6. Colon On-a-Plate™

The Colon-on-a-plate^™^ system is a high-throughput miniaturized version of the short-term 48 h batch fermentation model and has proven its ability to provide detailed mechanistic insights into the interplay between test products and the human gut microbiome [29,30].

This study utilized fecal material from 8 healthy individual donors (aged 20–40 years) who were on a typical Western diet, had no history of chronic gastrointestinal disease, and did not use antibiotics during the 4 months preceding stool collection. Half of the donors were male, and half female. Fecal materials were collected, stored, and used, as approved by the Ethics Committee of the University Hospital Ghent (reference number ONZ-2022-0267). An informed consent form was available for all donors.

At the start of the experiment, the wells of the Colon-on-a-plate^®^ (24-well plates with 10.4 mL volume-capacity; Thomson, Oceanside, BC, Canada) were filled with 6.2 mL of a carbohydrate-depleted nutritional medium (pH 6.5; PD001; ProDigest, Gent, Belgium). A single dose (0.8 mL) of pooled undigested fraction (pooled triplicates obtained from upper GIT digestion) for each bread recipe (BR1, BR2, BR3, BR4) or blank medium (water dialyzed as in the upper GIT digestion model) was added to each well to obtain a total volume of 7 mL. This theoretically resulted in a concentration of 8 g/L dry weight in each well (not accounting for product absorption during dialysis). However, the actual product concentrations in the wells were significantly lower given the amount of product absorption during dialysis. Finally, 10% (*v*/*v*) cryopreserved fecal inoculum containing 7.5% (*w*/*v*) fecal material from 1 of 8 healthy human donors was added to each well and served as the microbial source. Each step of this process was performed in an anaerobic chamber where oxygen levels were carefully monitored. Plates were incubated in an anaerobic atmosphere at 37 °C for 48 h. Following this, the samples were collected for the analysis of microbiota metabolic activity and composition. Each condition was tested in a single technical repetition using each of the 8 donors (i.e., 8 biological replicates per test condition).

### 2.7. Microbiota Metabolic Activity and Composition

The pH of the Colon-on-a-plate™ supernatants was determined using a Senseline F410 pH meter (ProSense, Oosterhout, The Netherlands). The levels of SCFAs (acetate, propionate, and butyrate, caproate, and valerate), and branched SCFAs (isobutyrate, isovalerate, and isocaproate) were measured using methods described by De Weirdt et al. [31]. Lactate was measured using a commercially available enzymatic assay kit (R-Biopharm, Darmstadt, Germany) according to the manufacturer’s instructions.

To assess microbiota composition changes between different BR digesta, shallow shotgun sequencing analysis was performed. For this, DNA was isolated as previously described by Van den Abbeele et al. [27], with a minor modification. A bead-beating step (glass beads, 0.1 mm; Carl Roth) was included using the NGS PreMax Homogenizer device (BioSPX, Abcoude, The Netherlands), performing the homogenization procedure twice for 40 s at 6 m/s, with 5 min in between. Following extraction, DNA concentrations were determined using the Quant-iT kit (Thermo Fisher Scientific, Waltham, MA, USA) according to the manufacturer’s instructions.

DNA libraries were prepared using the Nextera XT DNA Library Preparation Kit (Illumina, San Diego, CA, USA) and IDT Unique Dual Indexes with a total DNA input of 1 ng. Genomic DNA was fragmented using a proportional amount of Illumina Nextera XT fragmentation enzyme. Unique dual indexes were added to each sample, followed by 12 cycles of PCR to construct the libraries which were purified using AMpure magnetic Beads (Beckman Coulter, Brea, CA, USA) and eluted in QIAGEN EB buffer. DNA libraries were quantified using a Qubit 4 fluorometer and Qubit™ dsDNA HS Assay Kit (Thermo Fisher Scientific).

DNA libraries were pooled together proportionally based on target read depth. A quality check of the pooled library using Tapestation (Agilent Technologies, Les Ulis, France) examined the fragment size and concentration of the pool. Then, the pooled library was diluted and denatured using a standard protocol. Paired-end sequencing (2 × 150 bp) was performed using an Illumina HiSeq4000. The samples had an average read depth of 4.99 million reads.

The taxonomic classification of the unassembled sequencing reads was analyzed directly using the CosmosID-HUB Microbiome Platform (CosmosID Inc., Germantown, MD, USA) for multi-kingdom microbiome analysis and quantification of relative abundances, as previously described [32,33,34,35]. The annotated and normalized dataset was analyzed using CosmosID tools and exported to R (v.4.0.3) for multivariate analysis.

Total cell counts in each sample were determined using a BD Accuri C6 Plus Flow Cytometer (BD Biosciences, Franklin Lakes, NJ, USA), using the high flow rate setting with a threshold of 700 on the SYTO channel. To account for differences in bacterial biomass across the samples, relative abundances of each population in a sample were multiplied with the total cell count obtained by flow cytometry for a given sample to convert proportional values obtained using shotgun sequencing to absolute quantities.

### 2.8. Statistical Analysis

Pairwise comparisons of nutrient content between BRs (BR1 vs. BR2, effect of flour in 30% sourdough bread; BR3 vs. BR4, effect of flour in 0% sourdough bread; BR1 vs. BR3, effect of sourdough in white flour bread; BR2 vs. BR4, effect of sourdough in whole grain flour bread) and between blanks and BRs were performed using Dunn’s tests, with an adjustment of *p*-value for multiplicity testing with the Benjamani–Hochberg method. Differences were considered significant if *p* < 0.05.

Differences in microbiota metabolite production were analyzed using paired two-sided *t*-tests; data points for each fecal donor were considered replicates for the paired *t*-tests to demonstrate significance across donors. Differences were considered significant if *p* < 0.05.

Alpha diversity was evaluated using the Chao1 (measure for species richness) and Shannon (measure for species richness and evenness) indexes. Beta diversity was evaluated using Principal Coordinates Analysis (PCoA) on the Bray–Curtis and Jaccard distance matrix to represent the clustering of each BR for the 8 individual donors on the genetic diversity plane.

Linear Discriminant Effect Size (LEfSe) analysis was conducted on the total sum-scaled taxonomic abundances at the species level [36]. Only features that met *p* ≤ 0.05 for the Kruskal–Wallis and Wilcoxon tests were shown in LEfSe plots. There were no restrictions for minimal linear discriminant analysis (LDA) scores, though it is generally considered that a score of ≥2 is considered biologically relevant. TreeclimbR analysis was also conducted [37] and shown using volcano plots, with a cut-off for statistical significance of *p* < 0.05 (or −log10 0.05 = 1.3 on the *y*-axis). Bacterial enrichments with a −log(*p*-value) > 1.3 were considered statistically significant. Taxa were classified into four different categories: (1) not significant and not biologically relevant (−2 < log2FC < +2, and −log10[*p*-value] < 1.3), (2) biologically relevant, but not statistically significant (log2FC < −2 or log2FC > +2, and −log10[*p*-value] < 1.3), (3) statistically significant, but not biologically relevant (−2 < log2FC < +2, and −log10[*p*-value] > 1.3), and (4) biologically and statistically significant (log2FC < −2 or log2FC > +2, and −log10[*p*-value] > 1.3).

Univariate statistics were performed using Prism 10 (Graphpad, San Diego, CA, USA). All other analyses were performed using R version 4.2.2 (The R Foundation for Statistical Computing, Vienna, Austria).

## 3. Results

### 3.1. Flour Characterization

The characteristics of the two wheat flour types used (white and whole grain from the same wheat origin and miller), including flour properties and mineral, carbohydrate, and amylase contents are shown in Table 1. As expected, the whole grain flour had higher levels of protein, ash, minerals, and amylase compared with the white flour, while the white flour tended to have higher levels of fermentable and damaged starch.

### 3.2. Bread Characterization Pre-Digestion

#### 3.2.1. Texture Profile Analysis

Breads prepared with whole grain flour had significantly higher TPA values for hardness, fracturability, gumminess, and chewiness and significantly lower values for springiness, cohesiveness, and resilience compared with their respective bread types (sourdough or no sourdough) prepared with white flour (BR1 vs. BR2 and BR3 vs. BR4; adjusted *p* < 0.001 for all) (Table 2). Sourdough breads had significantly higher TPA values for springiness, cohesiveness, and resilience compared with non-sourdough bread made with the same flour type (BR1 vs. BR3 and BR2 vs. BR4; adjusted *p* < 0.001 for all).

#### 3.2.2. Nutritional Composition

The nutritional composition of each BR is presented in Table 3. The levels of free P + Ca + Mg + Fe + Zn ± SD were 153.56 ± 5.22, 409.78 ± 19.11, 123.79 ± 8.93, and 201.55 ± 3.70 mg/100 g dry bread for BR1, BR2, BR3, and BR4, respectively, and the total P + Ca + Mg + Fe + Zn ± SD values were 235.58 ± 19.96, 511.62 ± 17.89, 224.70 ± 9.83, and 447.87 ± 40.61 mg/100 g dry bread. While the two breads made with whole grain flour (BR2 and BR4) had numerically higher levels of free and total minerals than the breads made with white flour (BR1 and BR3), statistical significance was not reached among the BRs.

Free carbohydrate levels (glucose + maltose + fructose + galactose) were significantly higher for BR3 (3.80 ± 0.25 mg/100 g dry bread) compared to BR1 (0.26 ± 0.13) (adjusted *p*-value, 0.013), and free maltose levels were significantly higher for BR3 compared to BR4 (2.49 ± 0.34 vs. 1.25 ± 0.10 mg/100 g dry bread; adjusted *p*-value, 0.014). There were no other significant differences in carbohydrate content among the BRs.

### 3.3. In Vitro Digestibility

#### 3.3.1. Mineral Release

**Figure 2:**
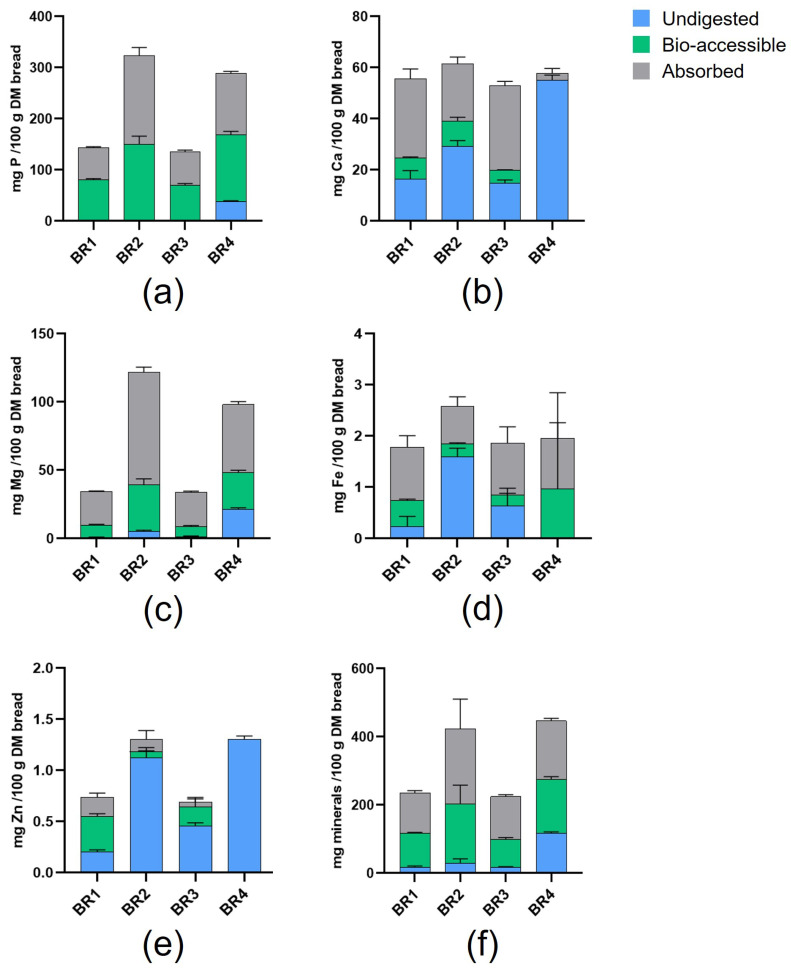
Mineral release from BRs following in vitro digestion. (**a**) P, (**b**) Ca, (**c**) Mg, (**d**) Fe, (**e**) Zn, (**f**) total minerals. Data are shown as mean ± standard deviation (*n* = 3). BR = bread recipe.

The levels of minerals released from BRs following in vitro digestion are shown in Figure 2. The BRs that used whole grain flour (BR2 and BR4) had numerically higher levels of digested (absorbed fraction + bio-accessible fraction) P and Mg compared with those using white flour (BR1 and BR3). In general, there were relatively high levels of undigested Ca, Fe, and Zn. The highest level of undigested minerals was reported for BR4.

#### 3.3.2. Carbohydrate Release

**Figure 3:**
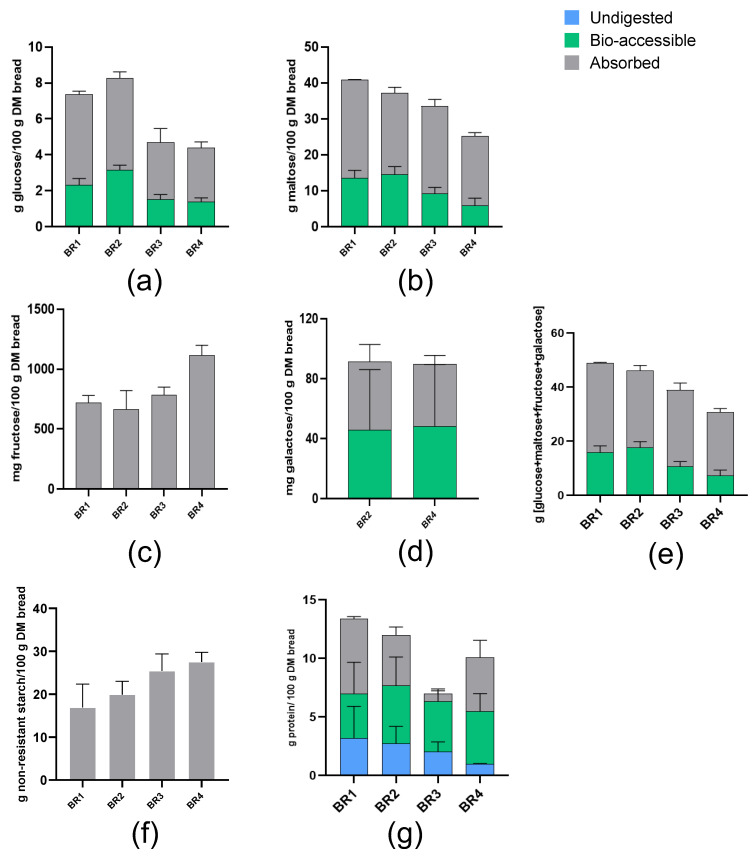
Carbohydrate and protein release from BRs following in vitro digestion. (**a**) Glucose, (**b**) maltose, (**c**) fructose, (**d**) galactose, (**e**) total carbohydrates, (**f**) non-resistant starch, (**g**) protein. BR1: white bread, 30% white flour sourdough + 0.5% yeast; BR2: whole grain bread, 30% whole grain flour sourdough + 0.5% yeast; BR3: white bread, 0% sourdough + 0.5% yeast; BR4: whole grain bread, 0% sourdough + 0.5% yeast (see Figure 1 for details). Data are shown as mean ± standard deviation (*n* = 3). BR = bread recipe.

Levels of carbohydrates released from BRs following in vitro digestion (absorbed fraction + bio-accessible fraction) are shown in Figure 3a–f. BRs that included sourdough (BR1 and BR2) tended to release higher levels of monomeric carbohydrates than non-sourdough recipes (BR3 and BR4), except for fructose which was highest for BR4. Non-resistant starch tended to be higher in BR3 and BR4 than in BR1 and BR2.

#### 3.3.3. Protein Release

The levels of protein released from BRs following in vitro digestion are shown in Figure 3g. Protein digestibility (absorbed fraction + bio-accessible fraction as compared to the total amount of protein within the BRs) was highest for BR4, followed by BR1, BR2, and BR3. However, no statistically significant differences were found for protein digestibility.

#### 3.3.4. Amino Acid Release

After normalizing the data to absorbed protein, BR3 released the highest amount of total free amino acids following digestion (absorbed fraction + bio-accessible fraction) (240.35 ± 42.28 mg/100 g digested bread); the values for the other BRs ranged between 28.56 ± 3.53 and 49.07 ± 3.85 (Table 4). BR3 also released the highest amounts of free branched-chain amino acids and essential amino acids (47.14 ± 7.56 mg/100 g digested bread and 107.87 ± 18.15 mg/100 g digested bread, respectfully) compared with the other BRs (range: 5.42 ± 0.73 to 9.59 ± 0.66 and 13.09 ± 1.57 to 21.52 ± 1.63 mg/100 g digested bread, respectively). Free branched-chain amino acid release was significantly higher with BR3 than BR4 (adjusted *p*-value, 0.009).

#### 3.3.5. Phytate Content

Phytate content was measured in post-digestion samples. The highest values were obtained with BR4, followed by BR2, BR3, and BR1, but there were no significant differences in the pairwise comparisons (Table 4).

### 3.4. Colonic Fermentation of the Undigested Fraction in the Colon-on-a-Plate™ Model

#### 3.4.1. Microbiota Metabolic Activity

**Figure 4:**
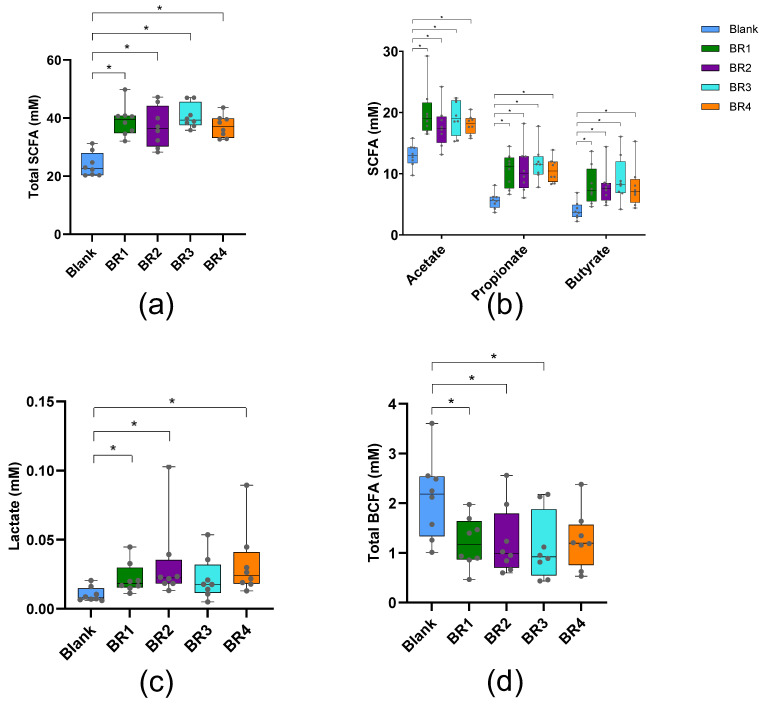
Microbiota metabolic activity in the Colon-on-a-plate™ model (undigested fraction) (**a**) total SCFA, (**b**) acetate, propionate, butyrate, (**c**) lactate, (**d**) total BCFA. Data from each of eight healthy donors were considered data replicates. Error bars represent standard deviation (*n* = 8 donors). Paired two-sided *t*-tests were used to determine significant differences between each condition versus the blank when accounting for the entire 48 h period. * *p* < 0.05. BCFA = branched chain fatty acid; BR = bread recipe; SCFA = short-chain fatty acid.

In the Colon-on-a-plate™ model, measures of saccharolytic fermentation (total SCFA [Figure 4a] and the individual main SCFAs acetate, propionate, and butyrate [Figure 4b]) were significantly increased from the blank 48 h after exposure to undigested BRs. Lactate levels were significantly increased with each BR (except BR3) compared with the blank (Figure 4c). Total BCFA, a measure of proteolytic fermentation, was significantly decreased with each BR (except BR4) compared with the blank (Figure 4d).

#### 3.4.2. Microbiota Composition

**Figure 5:**
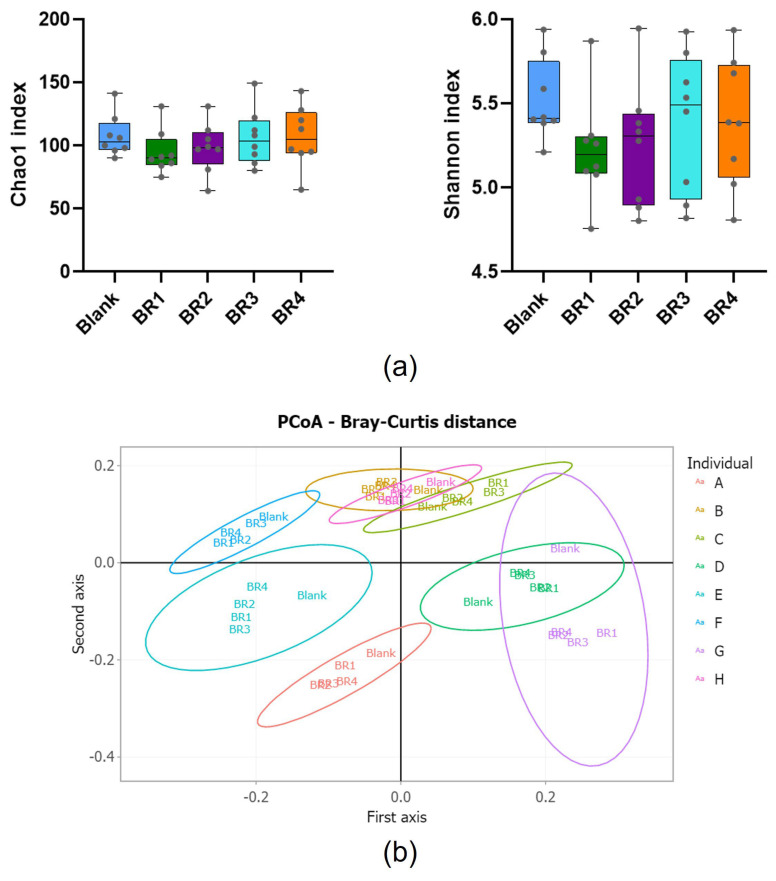
Analysis of the effects of the undigested fractions on microbiota (**a**) alpha (Chao1 and Shannon diversity indices) and (**b**) beta diversity (Principal Component Analysis) in the Colon-on-a-plate™ model. For Figure 5a, error bars represent standard deviation (*n* = 8 donors). For Figure 5b, this first plane displays 35% of the total variance (respectively, 20% for the first axis and 15% for the second axis). BR = bread recipe.

In general, the alpha diversity indices (Figure 5a) indicated that the microbiota diversity was quite stable following treatment with the different BRs. Beta diversity analysis also demonstrates a clustering of diversity for each donor, with a clear difference between the blank and the BRs and only minor differences among the different BRs (Figure 5b).

**Figure 6:**
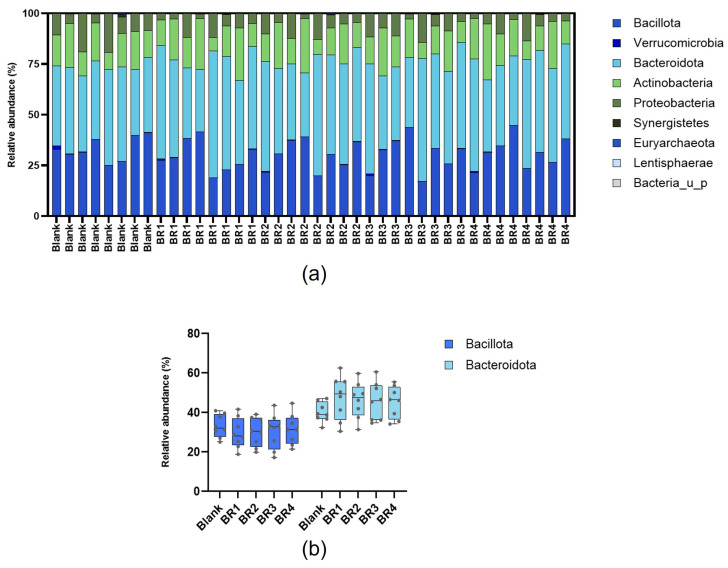
Relative abundance of gut microbiota following 24 h of fermentation (blank or undigested fraction) in the Colon-on-a-plate™ model (phylum level) for (**a**) all phyla (individual fecal donors, from donor 1 to 8 from left to right for blank or each bread recipe tested) and (**b**) predominant phyla (data represent the average of eight fecal donors, *n* = 8).

**Figure 7:**
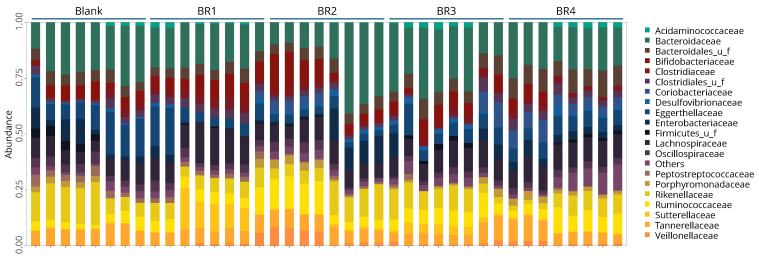
Relative abundance of gut microbiota following 24 h of fermentation (blank or undigested fraction) in the Colon-on-a-plate™ model (family level) (individual fecal donors from donor 1 to 8 from left to right for blank or each BR tested). BR = bread recipe.

At the phylum level, the intestinal microbiota composition in the Colon-on-a-plate™ model mainly consisted of Bacillota (i.e., Firmicutes) and Bacteroidota (i.e., Bacteroidetes) for all test conditions (blank, BR1, BR2, BR3, BR4) and all donors (Figure 6a). The relative abundance ranged from 20.0% to 44.8% for Bacillota and from 31.5% to 60.5% for Bacteroidota (Figure 6b). Furthermore, no clear differences in the relative abundances of different microbial families could be observed between the blank and any of the BRs (Figure 7).

**Figure 8:**
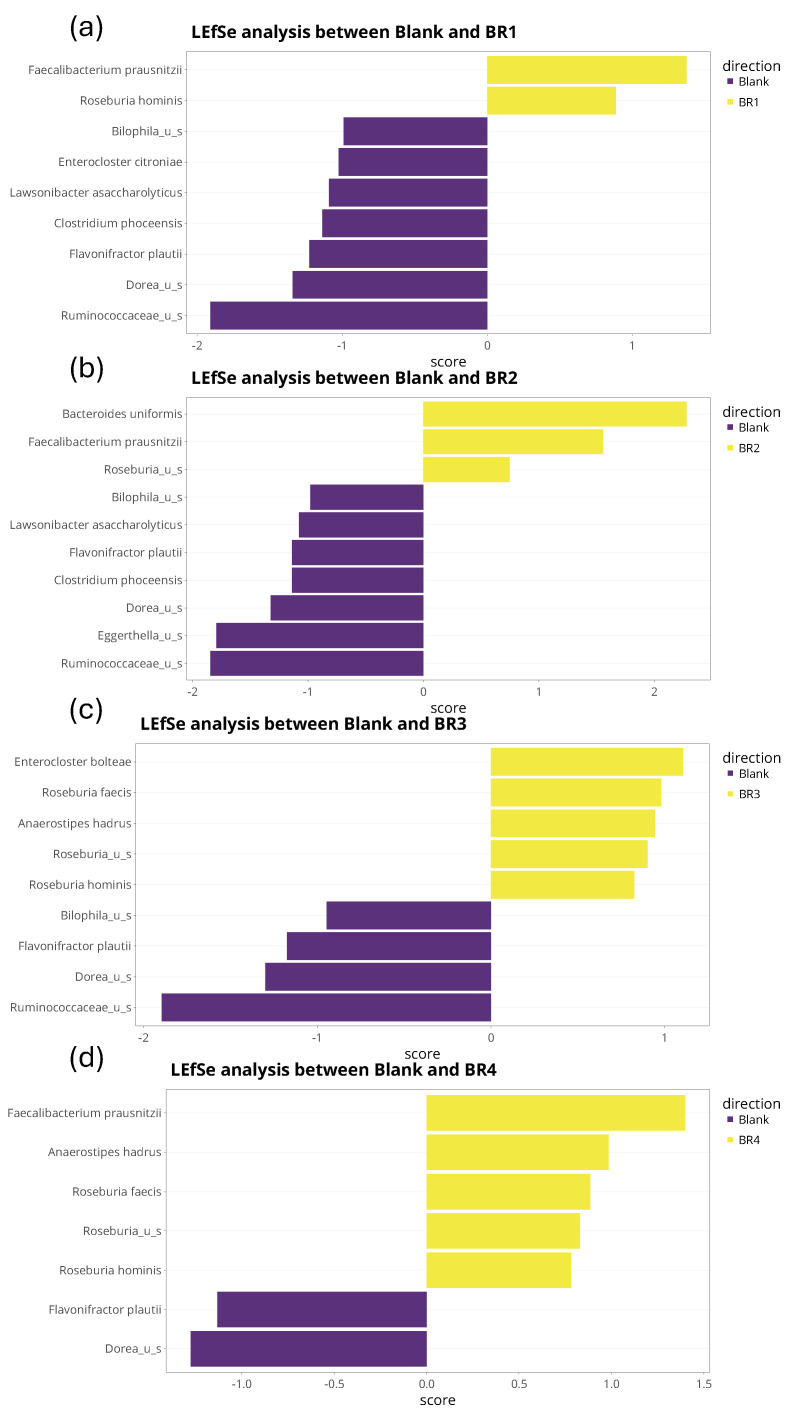
Differential abundance analysis (LEfSe) to identify differences in microbiota community composition (species level) at 24 h after the start of incubation with (**a**) undigested BR1, (**b**) undigested BR2, (**c**) undigested BR3, or (**d**) undigested BR4 versus blank. The analyses are based on relative abundance data (total sum scaling). The LEfSe bar plot shows significantly altered bacterial species between the blank and the indicated test product. Sections highlighted in yellow represent features that were significantly enriched by the undigested BR, while sections in purple were more abundant in the blank. The *x*-axis represents the LDA score (measure of effect size), with LDA scores of ±2 generally accepted as biologically relevant.

**Figure 9:**
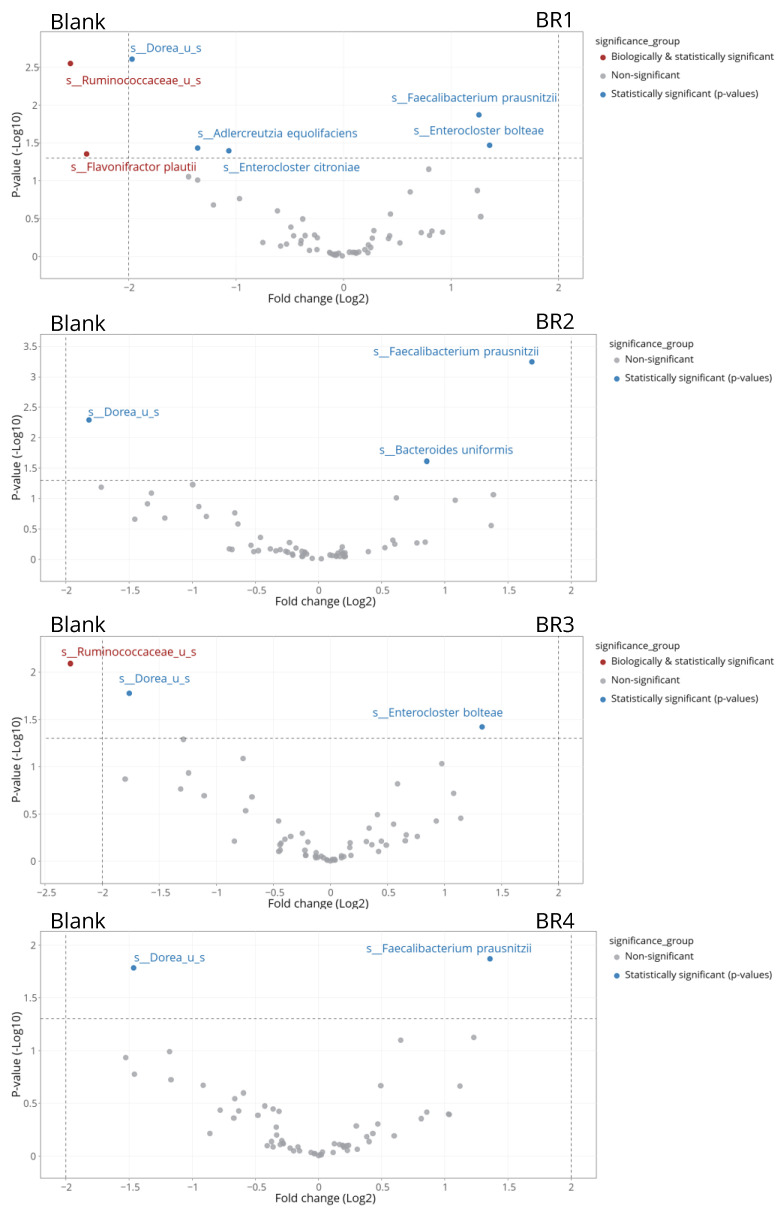
Differential abundance analysis (treeclimbR) to identify differences in microbiota community composition at 24 h after the start of incubation with undigested BR1, undigested BR2, undigested BR3, or undigested BR4 versus blank. The analysis is based on relative abundance data (total sum scaling). The scatter plot classifies taxa into four categories based on abundances in the compared conditions: neither biologically nor statistically significant (gray), biologically significant but not statistically significant (teal), statistically significant but not biologically significant (blue), or biologically and statistically significant (red).

**Figure 10:**
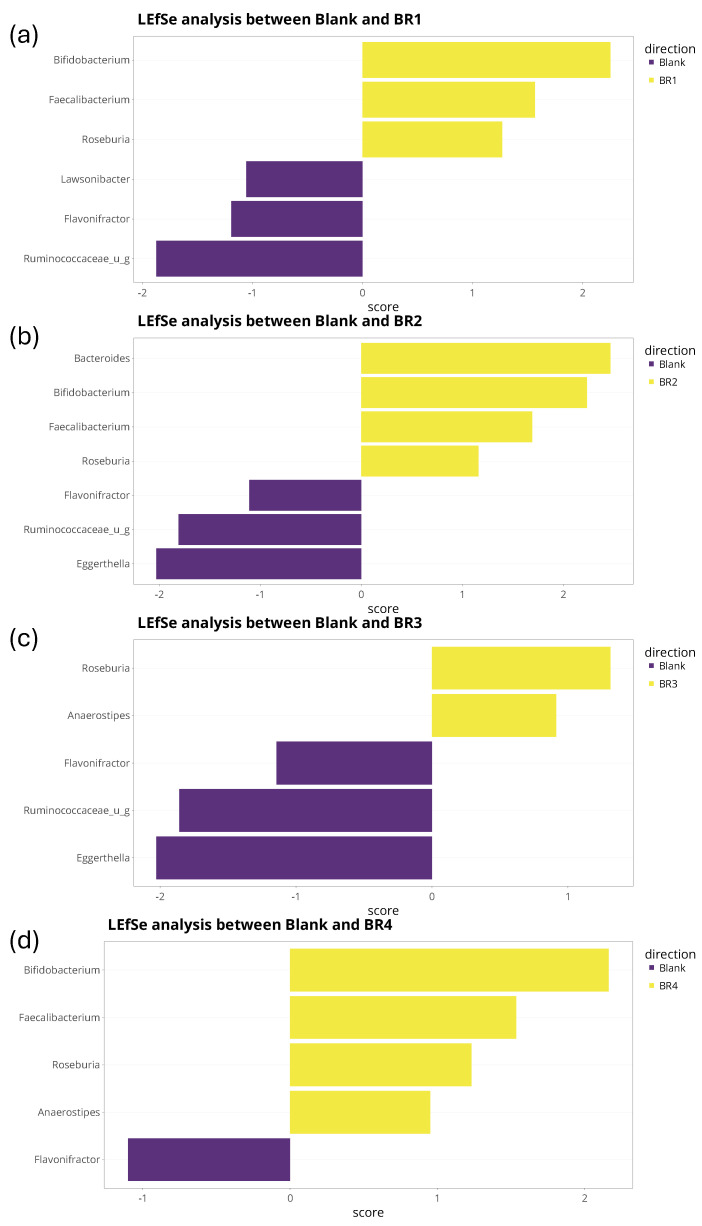
Differential abundance analysis (LEfSe) to identify differences in microbiota community composition (genus level) at 24 h after the start of incubation with (**a**) undigested BR1, (**b**) undigested BR2, (**c**) undigested BR3, or (**d**) undigested BR4 versus blank. The analyses are based on relative abundance data (total sum scaling). The LEfSe bar plot shows significantly altered bacterial genera between the blank and the indicated test product. Sections highlighted in yellow represent features that were significantly enriched by the undigested BR, while sections in purple were more abundant in the blank. The *x*-axis represents the LDA score (measure of effect size), with LDA scores of ±2 generally accepted as biologically relevant.

treeclimbR and LEfSe analysis were performed at the bacterial species and genus level. As confirmed following LEfSe analysis (Figure 8), treeclimbR analysis demonstrated a significant increase in the abundance of *Faecalibacterium prausnitzii* with BR1, BR2, and BR4 compared with the blank and a significant decrease in the abundance of *Dorea* unidentified species for all four BRs compared with the blank (Figure 9). Additionally, LEfSe analysis revealed an increased abundance of *Roseburia* spp. and a decreased abundance of *Flavonifractor plautii* following BR supplementation compared with the blank. Some changes observed in the treeclimbR and LEfSe analyses were unique for a given BR. For example, the undigested fraction of BR3 enabled an increased abundance of *Enterocloster bolteae* compared with the blank, which was not observed with the other BRs (Figure 8 and Figure 9), and *Ruminococcaceae* unidentified species were biologically and significantly decreased with BR1 and BR3 compared with the blank (Figure 8 and Figure 9). When comparing BR1 to BR2, BR3 to BR4, BR1 to BR3, and BR2 to BR4, no significant differences were observed. While these results were confirmed for the analysis at the bacterial genus level, genus-level comparisons did indicate the enrichment of *Bifidobacterium* associated with BR1, BR2, and BR4, but not BR3 (Figure 10). As this was not observed at the bacterial species level, the enriched *Bifidobacterium* species differed amongst the donors.

## 4. Discussion

Overall, this study demonstrated major differences in the texture of breads made from white versus whole grain flour, and those made using sourdough or yeast bread, with minor differences in nutritional content and digestibility among white breads (BR1 and BR3) or among whole grain breads (BR2 and BR4). The upper GIT digestion experiments demonstrated that a considerable proportion of the starch and protein content was digested at the end of the process, suggesting that all BRs resulted in good digestibility despite minor differences among the BRs. Further, colonic fermentation of the undigested fraction demonstrated effects on microbial metabolic activity, with increased SCFA production and decreased BCFA production relative to a blank control.

As expected, flour type (white vs. whole grain) and fermentation agent/time (sourdough bread vs. yeast bread) impacted the bread TPA (Table 2). Whole grain (vs. white) breads had lower springiness, cohesiveness, and resilience values, while sourdough breads (vs. yeast breads) had higher levels, irrespective of flour type [38,39,40]. The main differences observed between the recipes derived from the flour type rather than the type of ferment (yeast vs. sourdough fermentation). The higher hardness, fracturability, and gumminess of the bread samples prepared with whole grain flour and their respective lower springiness and resilience indicate the harder texture of the bread samples prepared with whole grain flour and the difficulty of these breads to spring back and regain their original texture and height after they compressed.

Levels of free and total minerals were numerically higher with whole grain versus white breads (Table 3), and pre-digested whole grain breads had the highest levels of digested minerals (absorbed + bio-accessible) (Figure 2), which is associated with the flour composition rather than the breadmaking process. Pre-digested whole grain breads (BR2 and BR4) had numerically higher levels of insoluble minerals, indicating entrapment in the remaining undigested food bolus. When considering minerals with low abundance (e.g., Fe and Zn), their levels in the absorbed and bio-accessible fractions were highly product-dependent. Sourdough fermentation appeared to increase the proportion of free to total mineral compared with yeast bread fermentation. Along these lines, the greatest level of carbohydrate digestion was observed for BR1 and BR2 (Figure 3e), suggesting that carbohydrate digestion was enhanced by the microbial activity that occurs with longer fermentation times and/or an increased amount of fermentation agent in the sourdough breadmaking process. Protein digestibility was greatest for BR4, though BR1 had the highest protein absorption rate, which indicates that a longer fermentation time with the inclusion of sourdough may improve the bioaccessibility of free amino acids (Figure 3g). Phytate was present and released in all BRs during upper GIT passage, with the whole grain recipes (BR2 and BR4) showing higher levels of total phytate compared with the white flour recipes (BR1 and BR3) (Table 4), suggesting that phytate was probably entrapped in the undigested food bolus upon using whole grain flour. Mineral, carbohydrate, and protein digestion were thus likely impacted somewhat by differences in the enzymatic (white vs. whole grain flour) and microbial ecology (sourdough vs. yeast bread) in the different BRs [38,39,40].

These findings are important given the different beneficial effects of nutrients on the human body. For example, Fe is the most abundant trace element in the body, followed by Zn, with both being critical for human health. Fe deficiency is the most common type of anemia [41], and Zn deficiencies are linked to reduced learning ability, worse Fe deficiency, and increased risk of cancer and infection [42,43]. Given the importance of these and other minerals, both the level and digestibility of minerals in bread are important considerations to optimize the nutritional profile. Furthermore, the intake and digestion of macromolecules, such as proteins, are also considered important for human health. However, the protein quality of wheat bread is poor, given that wheat flour has low levels of essential amino acids, most notably lysine [44]. The effects the breadmaking process can have on the digestibility of proteins are therefore important additional parameters to consider when characterizing the nutritional profile from wheat breads.

This study found that in vitro colonic fermentation of the undigested bread fraction following in vitro digestion resulted in the release of SCFAs (mainly acetate, propionate, butyrate) (Figure 4a), which are beneficial to human health in that they improve the integrity of the intestinal barrier, play a role in immune system function, and are involved in regulating inflammatory responses [45,46]. Attributed to differences in gut microbiota composition amongst individuals, detecting consistent microbial shifts across a population is typically more challenging. Regardless, some consistent findings were made across donors upon treatment with the BR. One interesting finding was that BR1, BR2, and BR4 strongly enriched *Bifidobacterium*, generally believed to benefit gut health. These products’ bifidogenic effects may have co-enriched the butyrate-producing *Faecalibacterium prausnitzii* through cross-feeding interactions. Indeed, *F. prausnitzii* converts acetate, produced by bifidobacteria, into butyrate. Interestingly, it was demonstrated that each bread recipe stimulated the growth of butyrate-producing bacteria, including *Roseburia* (each BR) and *Anaerostipes*, *with* the latter being exclusively attributed to the yeast BRs (BR3 and BR4). Finally, the enrichment of *Bacteroides uniformis* was exclusively observed for BR2.

In general, no significant differences were observed between the different BRs, which would indicate the minor impacts of flour type, fermentation agent, and time. This contrasts with general beliefs, in which whole grain wheat is considered to promote colonic health compared to white flour. It is hypothesized that while whole grain bread contains a significant fraction of insoluble fiber, the fiber fraction in white bread dissolves more easily, making it better accessible to the colonic bacteria, thus similar outcomes are obtained following single dosing. Still, the importance of insoluble fiber to promote the growth of important butyrate-producers has been described for its benefits in maintaining intestinal homeostasis and protecting the host against inflammation-related intestinal diseases [47]. Unlike pure dietary fiber, bread represents a complex matrix composed of different dietary components that are digested along the GIT. As a result, the effect on the intestinal microbiota composition is expected to be more subtle as compared to an equivalent amount of pure fiber that, by definition, reaches the colon fully intact. From these perspectives, single administration may be too limited to reveal the impact of insoluble fiber in the different BRs on the studied fermentation parameters, and a long-term administration model might be better suited, simulating repeated administration.

## 5. Conclusions

This study found that the biggest differences in the BRs evaluated were most notable in the TPA and that differences in nutrient composition, both pre- and post-digestion, were generally minor among similar flour breads after side-by-side comparison between sourdough or yeast BRs. Based on the cost, availability, and high consumption level, wheat bread is an excellent vehicle for supplementing nutrients in the diet. Optimal methods of bread and flour enrichment can be best accomplished with cooperation among different industries involved in breadmaking, including those involved in food hygiene, environmental health, nutritionists, farmers, and bakers [2]. Furthermore, assessing both the nutritional composition as well as the nutrient digestibility and colonic fermentation are important aspects to consider to fully characterize the nutritional profile and potential of different BRs.

## Figures and Tables

**Table 1:** Wheat flour characterization from a single batch of commercial flour used in this study.

	White Flour	Whole Grain Flour
Flour properties		
Moisture, % per 100 g flour	13.0	12.7
Protein, % per 100 g flour	11.45	12.6
Ash, % per 100 g flour	0.68	1.58
Falling number	391	356
Water absorption, %	56.6	63.1
Mineral content, ppm		
Potassium	1870	4160
Calcium	228	364
Magnesium	258	966
Iron	10.5	28.7
Carbohydrates, %		
Sucrose	0.3	0.5
Maltose	0.7	0.4
Glucose and fructose	<0.2	<0.2
Fermentable starch	67.8	58.6
Damaged starch	4.7	3
Amylase, U/g		
Ceralpha pH 5.2	0.18	0.24
Ceralpha pH 4.0	0.01	0.02
Betamyl pH 5.2	16.9	20.2
Betamyl pH 4.0	13.3	17.4

**Table 2:** Texture profile analysis.

Parameter	Bread Recipe				Adjusted *p*-Value			
	BR1	BR2	BR3	BR4	BR1 vs. BR2	BR3 vs. BR4	BR1 vs. BR3	BR2 vs. BR4
Hardness (g)	806.57 ± 172.11	2053.80 ± 397.91	848.81 ± 228.39	2674.17 ± 222.27	<0.001	<0.001	0.737	0.006
Fracturability (g)	311.68 ± 79.29	468.85 ± 121.86	334.63 ± 95.28	464.20 ± 141.57	<0.001	<0.001	0.505	0.805
Springiness (%)	95.0 ± 1.0	91.3 ± 1.1	93.6 ± 1.0	85.4 ± 14.1	<0.001	<0.001	< 0.001	0.003
Cohesiveness (%)	72.6 ± 3.3	65.8 ± 3.4	65.6 ± 3.9	57.8 ± 1.2	<0.001	<0.001	< 0.001	<0.001
Gumminess (-)	579.97 ± 101.67	1339.75 ± 211.66	549.31 ± 123.40	1544.85 ± 119.73	<0.001	<0.001	0.504	0.072
Chewiness (-)	550.45 ± 93.94	1222.66 ± 189.68	513.77 ± 113.47	1316.67 ± 212.55	<0.001	<0.001	0.462	0.462
Resilience (%)	43.0 ± 3.2	36.1 ± 3.2	35.4 ± 2.9	27.4 ± 1.2	<0.001	<0.001	<0.001	<0.001

Data are shown as mean ± standard deviation (*n* = 3). BR = bread recipe.

**Table 3:** Nutritional composition of BRs.

Parameter	Bread Recipe				Adjusted *p*-Value			
	BR1	BR2	BR3	BR4	BR1 vs. BR2	BR3 vs. BR4	BR1 vs. BR3	BR2 vs. BR4
Micronutrients								
Minerals (mg/100 g dry bread)								
Free P	82.95 ± 6.36	266.27 ± 14.62	74.58 ± 6.63	151.22 ± 2.56	0.094	0.14	0.571	0.411
Total P ^a^	143.01 ± 16.28	324.19 ± 11.04	135.23 ± 6.52	288.67 ± 21.59	0.07	0.179	0.734	0.411
Free Ca	35.82 ± 3.47	27.23 ± 0.43	19.78 ± 0.47	9.34 ± 0.56	0.308	0.308	0.083	0.083
Total Ca ^a^	55.79 ± 1.44	61.50 ± 7.92	53.10 ± 0.64	56.93 ± 9.44	0.497	0.411	0.411	0.411
Free Mg	34.00 ± 2.18	115.20 ± 4.33	29.18 ± 1.93	40.72 ± 0.64	0.083	0.083	0.308	0.308
Total ^a^ Mg	34.26 ± 2.37	122.03 ± 2.04	33.86 ± 2.28	98.13 ± 9.26	0.051	0.226	0.91	0.411
Free Fe	0.15 ± 0.07	0.25 ± 0.02	0.12 ± 0.003	0.17 ± 0.10	0.348	0.821	0.821	0.348
Total ^a^ Fe	1.78 ± 0.45	2.59 ± 0.63	1.87 ± 0.51	2.93 ± 0.62	0.226	0.226	0.821	0.821
Free Zn	0.64 ± 0.01	0.83 ± 0.02	0.14 ± 0.02	0.09 ± 0.004	0.308	0.308	0.308	0.009
Total ^a^ Zn	0.74 ± 0.09	1.30 ± 0.17	0.66 ± 0.08	1.21 ± 0.23	0.108	0.108	0.734	0.734
Free P + Ca + Mg + Fe + Zn	153.56 ± 5.22	409.78 ± 19.11	123.79 ± 8.93	201.55 ± 3.70	0.083	0.083	0.308	0.308
Total ^a^ P + Ca + Mg + Fe + Zn	235.58 ± 19.96	511.62 ± 17.89	224.70 ± 9.83	447.87 ± 40.61	0.070	0.179	0.734	0.411
Macronutrients								
Carbohydrates (mg/100 g dry bread)								
Nonresistant starch	49.19 ± 5.70	47.62 ± 1.53	53.00 ± 1.46	49.90 ± 0.95	0.343	0.365	0.343	0.343
Free glucose	0.15 ± 0.06	0.25 ± 0.02	0.24 ± 0.10	0.35 ± 0.09	0.226	0.308	0.226	0.308
Free maltose	0.11 ± 0.19	n.d.	2.49 ± 0.34	1.25 ± 0.10	–	0.014	0.178	–
Free fructose	n.d.	0.95 ± 0.25	1.06 ± 0.11	1.54 ± 0.23	–	0.051	0.074	–
Free galactose	n.d.	0.05 ± 0.04	n.d.	0.05 ± 0.04	–	1.000	–	–
Free glucose + maltose + fructose + galactose	0.26 ± 0.13	1.25 ± 0.25	3.80 ± 0.25	3.19 ± 0.38	0.411	0.428	0.013	0.411

^a^ Free + insoluble. Data are shown as mean ± standard deviation (*n* = 3). BR = bread recipe.

**Table 4:** Amino acid release (normalized to the absorbed protein content) from and phytate content (absorbed fraction) of BRs following in vitro digestion.

	Bread Recipe				Comparison *p*-Values			
					BR1 vs. BR2	BR3 vs. BR4	BR1 vs. BR3	BR2 vs. BR4
	BR1	BR2	BR3	BR4	adj	adj	adj	adj
AA category (mg/100 g digested bread)								
Free BCAA	6.84 ± 0.29	9.59 ± 0.66	47.14 ± 7.56	5.42 ± 0.73	0.31	0.009	0.055	0.055
Free EAA	15.13 ± 0.76	21.52 ± 1.63	107.87 ± 18.15	13.09 ± 1.57	0.26	0.013	0.063	0.072
Total free AA	34.27 ± 1.75	49.07 ± 3.85	240.35 ± 42.28	28.56 ± 3.53	0.74	0.44	0.74	0.44
Phytate content	75.29 ± 10.68	277.97 ± 38.34	112.89 ± 58.69	661.66 ± 202.2	0.197	0.077	0.582	0.357

Data are shown as mean ± standard deviation (*n* = 3). AA = amino acid; BCAA = branched-chain amino acid; BR = bread recipe; EAA = essential amino acids.

## Data Availability

The original contributions presented in the study are included in the article, further inquiries can be directed to the corresponding author.
